# Master Blaster: an approach to sensitive identification of remotely related proteins

**DOI:** 10.1038/s41598-021-87833-4

**Published:** 2021-04-22

**Authors:** Chintalapati Janaki, Venkatraman S. Gowri, Narayanaswamy Srinivasan

**Affiliations:** 1 Molecular Biophysics Unit, Indian Institute of Science, Bangalore, 560012 India; 2 Centre for Development of Advanced Computing, Knowledge Park, Byappanahalli, Bangalore, 560038 India; 3 Present Address: Department of Chemistry, Auxilium College, Gandhinagar, Vellore, 632006 India

**Keywords:** Protein sequence analyses, Structural biology

## Abstract

Genome sequencing projects unearth sequences of all the protein sequences encoded in a genome. As the first step, homology detection is employed to obtain clues to structure and function of these proteins. However, high evolutionary divergence between homologous proteins challenges our ability to detect distant relationships. In the past, an approach involving multiple Position Specific Scoring Matrices (PSSMs) was found to be more effective than traditional single PSSMs. Cascaded search is another successful approach where hits of a search are queried to detect more homologues. We propose a protocol, ‘Master Blaster’, which combines the principles adopted in these two approaches to enhance our ability to detect remote homologues even further. Assessment of the approach was performed using known relationships available in the SCOP70 database, and the results were compared against that of PSI-BLAST and HHblits, a hidden Markov model-based method. Compared to PSI-BLAST, Master Blaster resulted in 10% improvement with respect to detection of cross superfamily connections, nearly 35% improvement in cross family and more than 80% improvement in intra family connections. From the results it was observed that HHblits is more sensitive in detecting remote homologues compared to Master Blaster. However, there are true hits from 46-folds for which Master Blaster reported homologs that are not reported by HHblits even using the optimal parameters indicating that for detecting remote homologues, use of multiple methods employing a combination of different approaches can be more effective in detecting remote homologs. Master Blaster stand-alone code is available for download in the supplementary archive.

## Introduction

With the advent of genome sequencing projects number of sequences in the public databases is increasing exponentially^1^. Analysis of sequence databases of proteins suggests that there are a large number of uncharacterized proteins with no information on function, structure, post-translational modifications, protein–protein interactions, protein-nucleic acid interactions and protein-small molecule interactions^2^. In the current release of PFAM, a database of protein families, there are 4244 domains of unknown function (DUFs) that correspond to 23% of the domains^3^. Unfortunately, as the sizes of such sequence databases are increasing in a rapid pace, the gap in our understanding of above mentioned attributes of proteins are also widening. While multiple experimental studies are required for a detailed and complete understanding of the molecular and mechanistic basis of protein action and regulation, computational approaches can help to arrive at reasonable initial ideas on the functions, structures and other features of proteins^4^.

Homologous proteins are often known to adopt same or similar structure and function. However the problem with recognition of homologous or related proteins is high sequence divergence^5,6^. There are several known cases of homologous proteins sharing a sequence identity as low as between two unrelated proteins of entirely different structure and function.

Although many computational methods have been developed for remote homology detection over a few decades now^7^, often they are not always able to identify all the related proteins of a query especially when the relationship is distant characterized by very low sequence similarity. It is essential to develop new computational methods which address fundamental questions and arrive at reliable and more complete answers. Indeed, search results for a query sequence with two or more equally sensitive and successful search algorithms often do not match and one needs to consider union set of hits from multiple search programs.

Algorithms such as Needleman-Wunsch^8^, BLAST^9^, FASTA^10,11^ and Smith-Waterman^12^ have pioneered sequence alignment methods and formed foundation for profile-based methods which include PSI-BLAST^13^, HHsearch^14^, CS-BLAST^15^, HMMER3^16^, FFAS^17^, AlignHush^18^, HHblits^19^, HIPPI^20^ and many more. These methods employ sequence profiles represented either as Position Specific Scoring Matrix (PSSM)^13^ or as hidden Markov model (HMM). Profiles^21^ are built for every protein family and these profiles are used in sequence similarity searches.

Earlier studies from our and other laboratories have shown that Intermediate Sequence Search (ISS), multiple PSSMs and artificial sequences were found to be very effective in detecting remote homologues^22–26^. In Intermediate Sequence Search or cascade search approach every hit obtained as a result of search is used as a query to identify further related proteins. The simple principle used in such an approach is homologue of a hit should also be a homologue of the original query sequence. Cascading the search process enables traverse through the protein sequence space and consequently recognize distant homologues. Use of approaches outlined above involving combination of PSSM and HMM based methods were proven to be useful in enriching structural and functional annotation of complete genome of several organisms, for example, *Mycobacterium tuberculosis*^27^.

Benchmark studies of various homology inference tools^28,29^ have shown that most of the profile based methods perform better than single sequence search methods. To model long-range residue interactions and to overcome some of the limitations of HMM based methods, MRFalign detects homologues by modeling multiple sequence alignment (MSA) using Markov Random Fields (MRFs)^30^.

In this work we combine the power of two sensitive homology detection methods working in different principles thereby enhancing our ability to detect related proteins even better. One of the two developments which are combined in the present work is Cascade approach that feeds hits of a search process as queries in the next generation of searches^25^. In this approach every hit is explored for its potential to serve as intermediately related to two or more extremely distantly related proteins. The second development that is combined with the cascade approach is the use of multiple PSSMs (MulPSSM)^22,23^. Usually, a PSSM is generated from a MSA with one of the sequences involved in the MSA as the reference sequence. However, it has been shown that use of multiple PSSMs generated using a given MSA with different sequences as reference results in more sensitive remote homology detection^22,23^. The present work proposes a protocol referred as Master Blaster which uses the powerful principles built in Cascade and multiple PSSM approaches to result in improved homology search approach.

In the earlier developed cascade search methods, the intermediate sequence is used to increase the search space, but the sequence does not contain information on the conserved positions and insertions/deletions. In contrary, if the input is in the form of a profile, it can capture the information on not only the positions in which the residue is conserved but will also throw light on the position-specific insertions or deletions or mutations. Use of profiles instead of sequences has various applications like identification of substrate binding sites and secondary structure prediction^31^. Though Master Blaster adopts the concept of cascading, the methodology followed in using intermediate sequences as hits is different from what is followed in earlier developed method. Similarly, though MulPSSM concept is adopted, the approach in Master Blaster differs completely from the previous developments. In Master Blaster, the PSSMs are built internally and dynamically by PSI-BLAST from the multiple sequence alignment of the hits obtained in each iteration.

## Results and discussion

**Figure 1:**
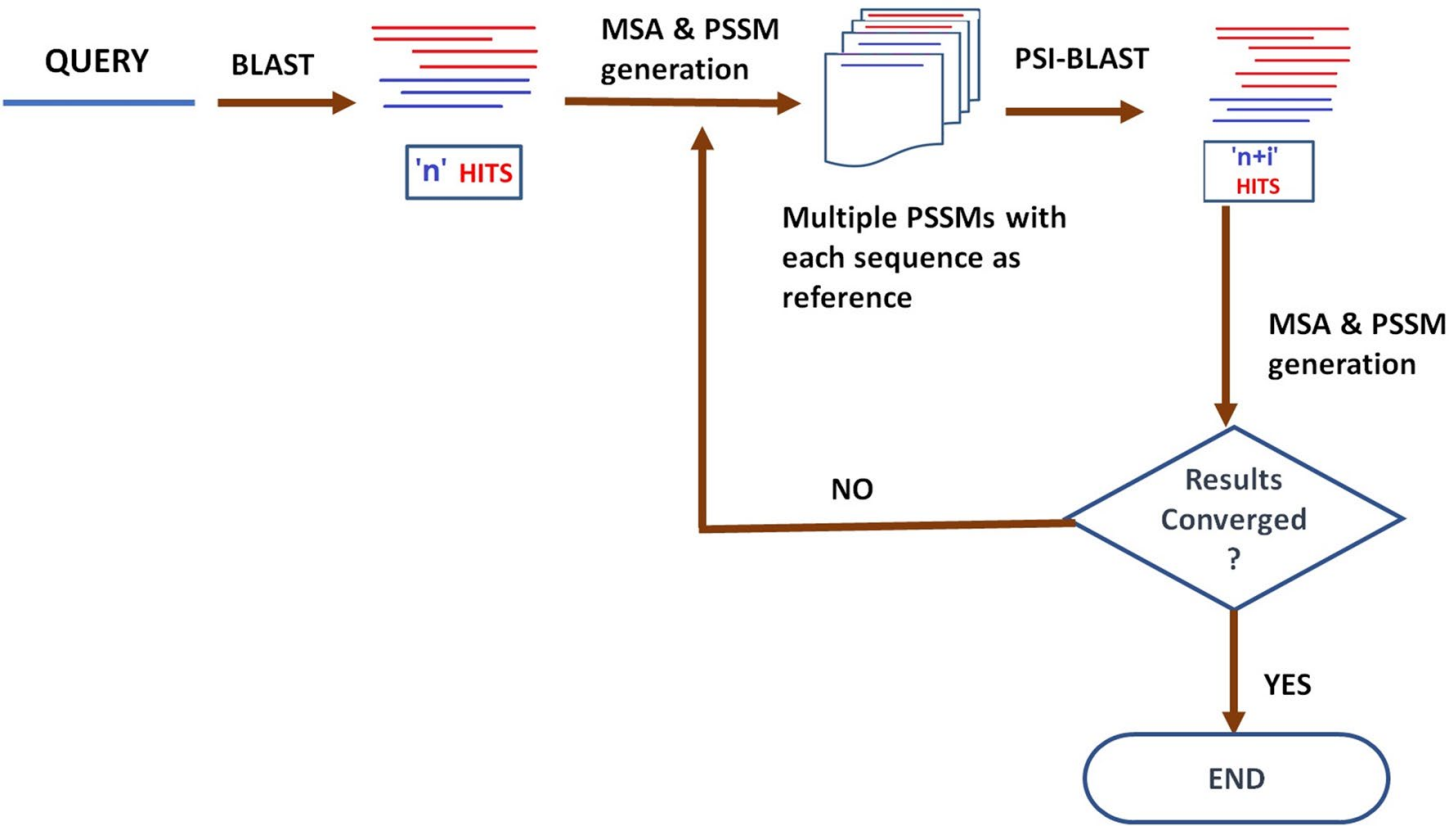
Flowchart of approach used for Master Blaster.

Details of the Master Blaster protocol proposed in this work are provided in Materials and Methods section and an outline of the protocol is shown in Fig. 1. Briefly, a sequence query is searched against a sequence database. Initial set of hit sequences are aligned and multiple PSSMs^13^ are generated internally by PSI-BLAST, using every one of the sequences used in the alignment as a reference sequence. This step takes the idea from multiple PSSM representations^23^ of a given multiple sequence alignment. Then multiple searches are made on the sequence database with each search using a PSSM, generated using PSI-BLAST, as the query. This step represents the cascade nature of the search process. Next, hits from these new set of searches are combined with the hits from previous generation and a new multiple sequence alignment and corresponding multiple PSSMs are generated using PSI-BLAST. These PSSMs are queried, and the search generation cycle continues (Fig. 1). The generation cycle is continued until the convergence or with a predetermined number of generations.

In this study, 2030 protein domains belonging to 153 SCOP folds (Structural Classification of Proteins)^32^ were used as query sequences and searched against searched against SCOP70 sequence dataset having 13,650 domain sequences (as discussed in Materials and Methods). These domains belonging to four major structural classes of SCOP, Alpha (a), Beta (b), Alpha and Beta (a/b) and Alpha and Beta (a + b). The same dataset is used for comparative assessment of PSI-BLAST^13^, Master Blaster and HHblits^19^, a hidden Markov model based method. No two domains in the SCOP70 database have more than 70% identity as the domains are already clustered based on their sequence identity. In all these searches if the fold of the query and fold of a hit are same according to their classification in SCOP, then such a hit is said to be a true positive. If the fold of the hit is not same as the fold of the query, then such a hit is considered false positive.

### Algorithm parameters for PSI-BLAST and Master Blaster

As algorithm parameters such as number of iterations, Expect value (E-value) and H-value criteria, % query coverage have influence on the results^13^, we executed different runs by changing these parameter values. In a practical situation of identifying distant homologues, it is important to consult results of multiple sequence search approaches working on different principles to conclude on the set of correct hits from the repertoire of all the hits from individual methods. Master Blaster and PSI-BLAST runs are executed using combination of different parameters (Table 1). We have set five iterations are for all the runs. Though many of the hits converge by second or third iteration, there are few query sequences for which search space is covered in fifth iteration or above.

**Table 1:** Program parameters used for comparative assessment studies of PSI-BLAST, Master Blaster and HHblits.

E-value	1e−2	1e−2	1e−3	1e−3
H-value	1e−2	1e−2	1e−3	1e−3
Query coverage	70%	60%	70%	60%
Number of iterations	5	5	5	5
Substitution matrix	BLOSUM 62	BLOSUM 62	BLOSUM 62	BLOSUM 62
BLASTClust identity	80%	80%	80%	80%
BLASTClust length coverage	70%	70%	70%	70%
Master Blaster iterations	5	5	5	5

### Algorithm parameters for HHblits

As the parameters used for Master Blaster may not be suitable for HHblits, we executed different runs of HHblits by considering different algorithm parameters. These include E-value, the number of iterations, MACT threshold parameter “mact” (Mact) values, and %query coverage (Table 2). MACT or Maximum accuracy threshold is based on an algorithm that controls the alignment greediness^19^ thus producing conservatives local alignments.

**Table 2:** Combination of parameters used to study the performance of HHblits.

S. no.	Number of runs	E-value	Mact value	%Query coverage
1	1	10	0.6	70
2	1	10	0.9	70
3	1	2	0.35	70
4	1	3	0.35	70
5	1	4	0.35	70
6	1	5	0.35	70
7	1	6	0.35	70
8	1	7	0.35	70
9	1	8	0.35	70
10	1	9	0.35	70
11	2	10	0.9	70
12	2	2	0.35	70
13	2	3	0.35	70
14	2	4	0.35	70
15	2	5	0.35	70
16	2	6	0.35	70
17	2	7	0.35	70
18	2	8	0.35	70
19	2	9	0.35	70
20	3	10	0.6	60
21	3	10	0.6	70
22	3	10	0.9	70
23	3	2	0.35	70
24	3	3	0.35	70
25	3	3	0.6	70
26	3	3	0.6	80
27	3	4	0.35	70
28	3	5	0.35	70
29	3	6	0.35	70
30	3	6	0.3	80
31	3	6	0.4	70
32	3	6	0.6	80
33	3	7	0.35	70
34	3	8	0.35	70
35	3	8	0.4	70
36	3	8	0.9	70
37	3	9	0.35	70
38	4	10	0.9	70
39	4	2	0.35	70
40	4	3	0.35	70
41	4	4	0.35	70
42	4	5	0.35	70
43	4	6	0.35	70
44	4	7	0.35	70
45	4	8	0.35	70
46	4	9	0.35	70
47	5	10	0.2	70
48	5	10	0.35	70
49	5	10	0.3	70
50	5	10	0.4	70
51	5	10	0.5	70
52	5	10	0.6	70
53	5	10	0.6	80
54	5	10	0.9	70
55	5	10	0.9	80
56	5	2	0.35	70
57	5	3	0.35	70
58	5	3	0.6	80
59	5	4	0.35	70
60	5	4	0.5	80
61	5	5	0.35	70
62	5	6	0.2	70
63	5	6	0.35	70
64	5	6	0.3	80
65	5	6	0.4	70
66	5	6	0.5	80
67	5	7	0.35	70
68	5	8	0.2	80
69	5	8	0.35	70
70	5	8	0.6	60
71	5	8	0.6	70
72	5	9	0.35	70

The influence of parameters for each tool on the homology detection is studied and the results are presented in Supplementary Table 1 and Supplementary Table 2 for Master Blaster and HHblits respectively.

### Assessment of Master Blaster by comparison to PSI-BLAST

Assessment of the new method was done using four performance measures, i.e., % Sensitivity or Recall, % Specificity, % Precision and % Error rate. Sensitivity is a measure of the ability to identify the true positives and Specificity is a measure of the ability to identify the true negatives. Higher the number of false negatives, lower is the sensitivity of the method and higher the number of false positives, higher is the error rate and lower specificity.

The results of Master Blaster using different parameter values are given in Supplementary Table 1. It can be seen from the results that, by increasing E-value from 1e−3 to 1e−2 and keeping the query coverage as 70%, the sensitivity increased as expected i.e., 0.32 to 0.36. With the change in query coverage from 70 to 60%, there is no improvement in the number of true positives. However, number of false positives increased as more shorter sequences are included in every iteration. With 70% query coverage and an E-value and H-value of 1e−2, the performance of Master Blaster is found to be optimum for this dataset.

**Figure 2:**
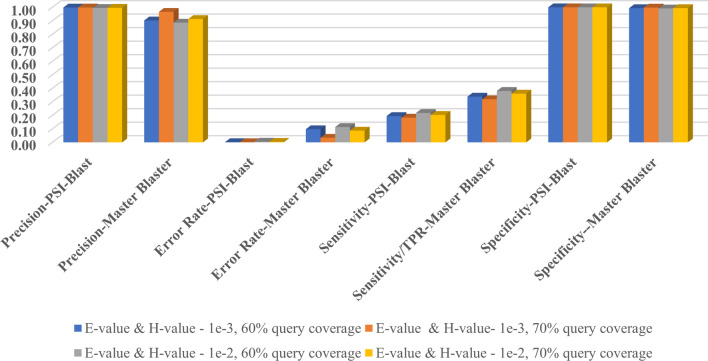
Comparative assessment of PSI-BLAST, and Master Blaster using different parameters.

The results of Master Blaster and PSI-BLAST are compared by considering the number of true positives reported by each method. Compared to PSI-BLAST, additional 50% true positives were reported by Master Blaster in five generations (Table 3). PSI-BLAST and Master Blaster results are comparable with respect to specificity i.e., 99% on an average. % Error rate is slightly higher for Master Blaster as there are hits reported from different folds which were added from third generation onwards. With 70% query coverage and a E-value and H-value of 1e−3, the error rate is 0.03 i.e., 3% (Supplementary Table 1). With increase in E-value and H-value from 1e−3 to 1e−2, the error rate increased from 3 to 9% indicating the statistical significance of E-value cutoff. However, compared to 1e−3, 4% improvement is observed in the number of true positives detected using e-value of 1e−2. From Fig. 2, it is evident that the average sensitivity using Master Blaster is higher compared to PSI-BLAST.

**Table 3:** Comparative performance of PSI-BLAST and Master Blaster.

Parameters	TP-PSIBLAST	TP-Master Blaster	%Improvement
E-value and H-value—1e−3, 60% query coverage	72,869	126,617	57.55
E-value and H-value—1e−3, 70% query coverage	68,463	119,345	57.37
E-value and H-value—1e−2, 60% query coverage	81,477	142,419	57.21
E-value and H-value—1e−2, 70% query coverage	76,036	135,025	56.31

Number of true positives (TP) reported by PSI-BLAST and Master Blaster and %Improvement.

### Cross superfamily, cross family and intra family connections within a fold

For any approach which aims to detect related proteins using their sequences, the most challenging task is to identify cross family (within a superfamily) and cross superfamily (within a fold) relationships. Detailed analysis of results behind Fig. 2 suggests that there are few folds for which the % sensitivity of Master Blaster is higher than that of PSI-BLAST due to improvement in cross superfamily, cross family and intra family connections (Supplementary Table 3). Therefore, it is important to compare inter-superfamily connections and inter-family connections using various methods. For each query, the hits belonging to a different superfamily as that of query are considered for an assessment of inter-superfamily connections. As seen in Supplementary Table 3, there are many cross superfamily, cross family and intra family connections made using Master Blaster. This means that the method is efficient enough to detect homologs from the same family or superfamily within the same fold. Protein pairs belonging to same superfamily but belonging to different families are typical benchmark for testing the ability of computational methods to identify remote homologues^39^.

An average of more than 10% improvement is observed with respect to cross superfamily connections, 35% improvement in cross family connections, more than 80% improvement in intra family connections. On the whole, an improvement of more than 50% is observed using Master Blaster. In folds such as P-loop containing nucleoside triphosphate hydrolases (c.37), PLP-dependent transferase-fold (c.67), alpha/beta-Hydrolases (c.69) having one superfamily (c.69.1), Immunoglobulin-like beta-sandwich (b.1), (TIM) beta/alpha-barrel (c.1), NAD(P)-binding Rossmann-fold domains (c.2), S-adenosyl-l-methionine-dependent methyltransferases (c.66), cross family and cross superfamily connections increased significantly with true positives getting added in every generation in Master Blaster. One such example is for the homologs detected from the triosephosphate isomerase (TIM) beta/alpha-barrel (SCOP fold c.1). TIM fold is one of the ancient folds and is found in 10% of enzymes as catalytic domains^33^. The proteins belonging to this fold have eight repeats of a β-strand and an α-helix, (β/α)8 and exhibit high functional diversity due to gene evolution and hence fall under 33 different superfamilies within the same fold. Using PSI-BLAST, 12 cross superfamily connections and 111 true positives are reported from this fold whereas using Master Blaster, 15 superfamilies and 800 additional true positives are reported. Three additional superfamilies are covered under cross superfamily connections. These includes Bacterial luciferase-like (c.1.16), Enolase C-terminal domain-like (c.1.11), and Metallo-dependent hydrolases (c.1.9).

Master Blaster is found to be more efficient in detecting remote homologues having less than 20% sequence identity. As there is more than one profile used for each family, protein sequence space increased drastically leading to increase in true positives in searches using Master Blaster.

### Assessment of Master Blaster by comparison to HHblits

HHblits^19^, a profile HMM based method is used widely for remote homology detection. It performs HMM-HMM alignment and is an extension of HHsearch^34^, a powerful hidden Markov based method for remote homology detection. We evaluated the performance of Master Blaster against HHblits using the same dataset used for comparison with PSI-BLAST i.e., 2030 domains from 153 SCOP folds as query sequences searched against SCOP70 database. The Master Blaster and HHblits results using different algorithm parameters are given in Supplementary Table 1 and Supplementary Table 2, respectively.

**Figure 3:**
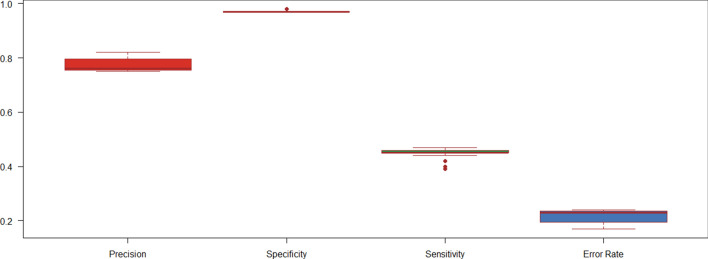
Sensitivity, Specificity, Precision, and Error Rate for HHblits runs using different E-value thresholds, number of iterations, and MACT values are given in the box plot.

For HHblits, with the change in parameters, the sensitivity values varied from 0.39 to 0.47, the relative error rate from 0.14 to 0.24, precision from 75 to 82%, and no significant change in specificity is observed (as seen from boxplot in Fig. 3). With MACT value greater than 0.5, there is an improvement in the precision and this is in concurrence with what has been reported by Remmert et al. in 2012^19^. With an E-value equal to or lower than 1e−8, Mact value above 0.5 and the number of iterations between 3 to 5, an optimal performance is observed i.e., higher sensitivity, higher precision, and with relatively lower error rate. By varying MACT value and keeping all the other parameters constant it is observed that there is significant difference in the number of true positives for few of the folds (Supplementary Table 4). For example, for the fold a.1 (Globin-like [46457] (2 superfamilies) the number of true positives using default MACT value 0.35 is 569 and using 0.9 it is 858. The results obtained using these values are considered as optimal and are used for further analysis and comparison with Master Blaster.

**Figure 4:**
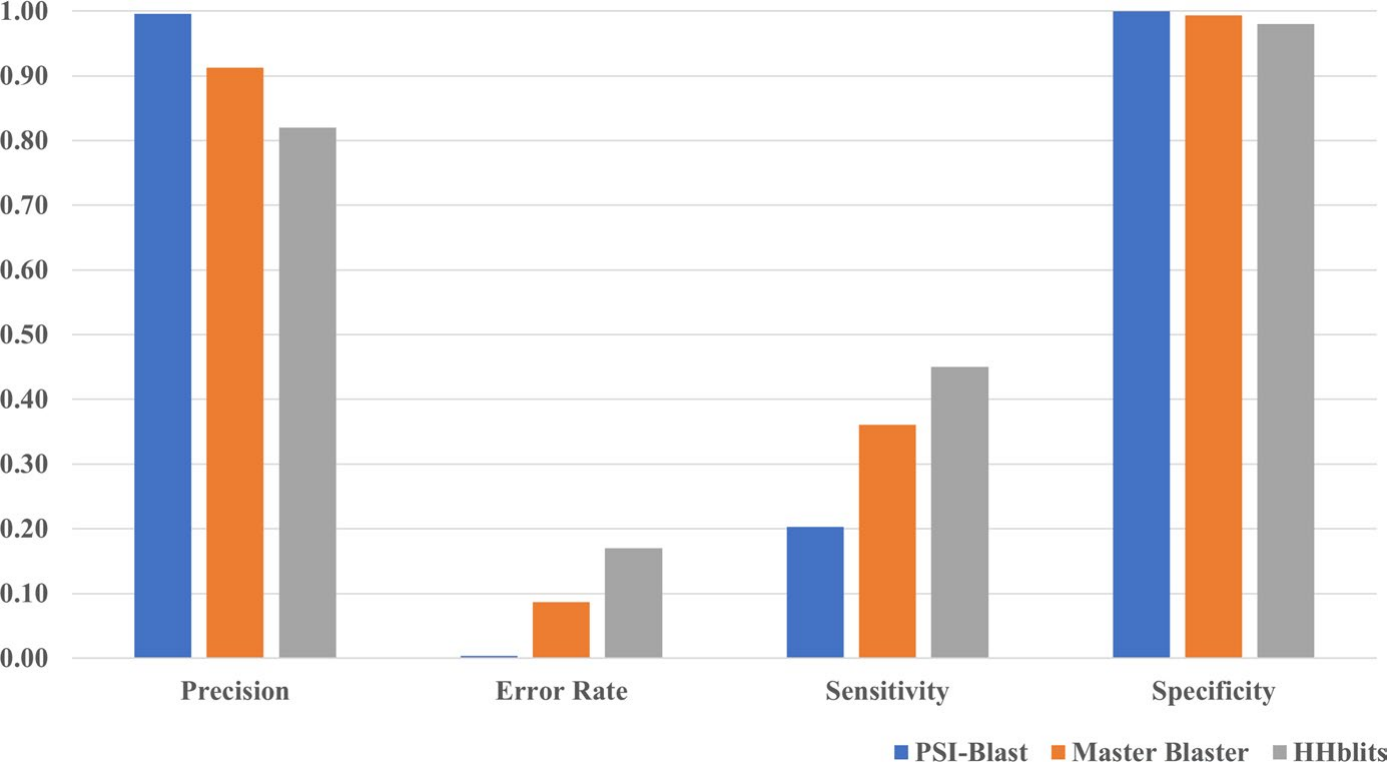
Comparative assessment of PSI-BLAST, Master Blaster and HHblits using Sensitivity, Specificity, Precision and Error rate as performance metrics. Performance of PSI-BLAST is represented in Blue, Master Blaster in Orange and HHblits in Grey color.

Comparative assessment of the methods is done using performance metrics such as Sensitivity, Specificity, Precision, and Error rate is given in Fig. 4. As seen in the Fig. 4, HHblits is found to be relatively more sensitive in identifying the remote homologs compared to PSI-BLAST and Master Blaster (using E-value of 10^–2^). At an E-value of 1e−2 it could be noticed that the performance of Master Blaster is close to HHblits (Supplementary Table 2) in recognizing the true positives. However, despite use of optimal parameter values, the number of false positives is significantly higher using HHblits compared to the other two approaches, leading to higher error rate and precision (Fig. 4).

We have analysed the true positive rate and fold coverage using each method for different SCOP superfamilies. Out of 153-folds, 36-folds had same fold coverage for Master Blaster and HHblits. For many of the superfamilies, the true positive rate is found to be twofold higher using HHblits compared to Master Blaster. At the same time, Master Blaster could detect many cross family connections that are not reported by HHblits suggesting that it is essential to use as many different methods as possible to search the protein space.

There are many queries for which hits are common between both the approaches and for some sequences results are unique to one particular approach. HHblits being a method based on hidden Markov models, it is more sensitive in detecting remote homologues compared to other two approaches but with a compromise on error rate as there are number of hits reported from folds and classes different from that of the query sequence (Fig. 4).

### Unique hits reported by Master Blaster

**Figure 5:**
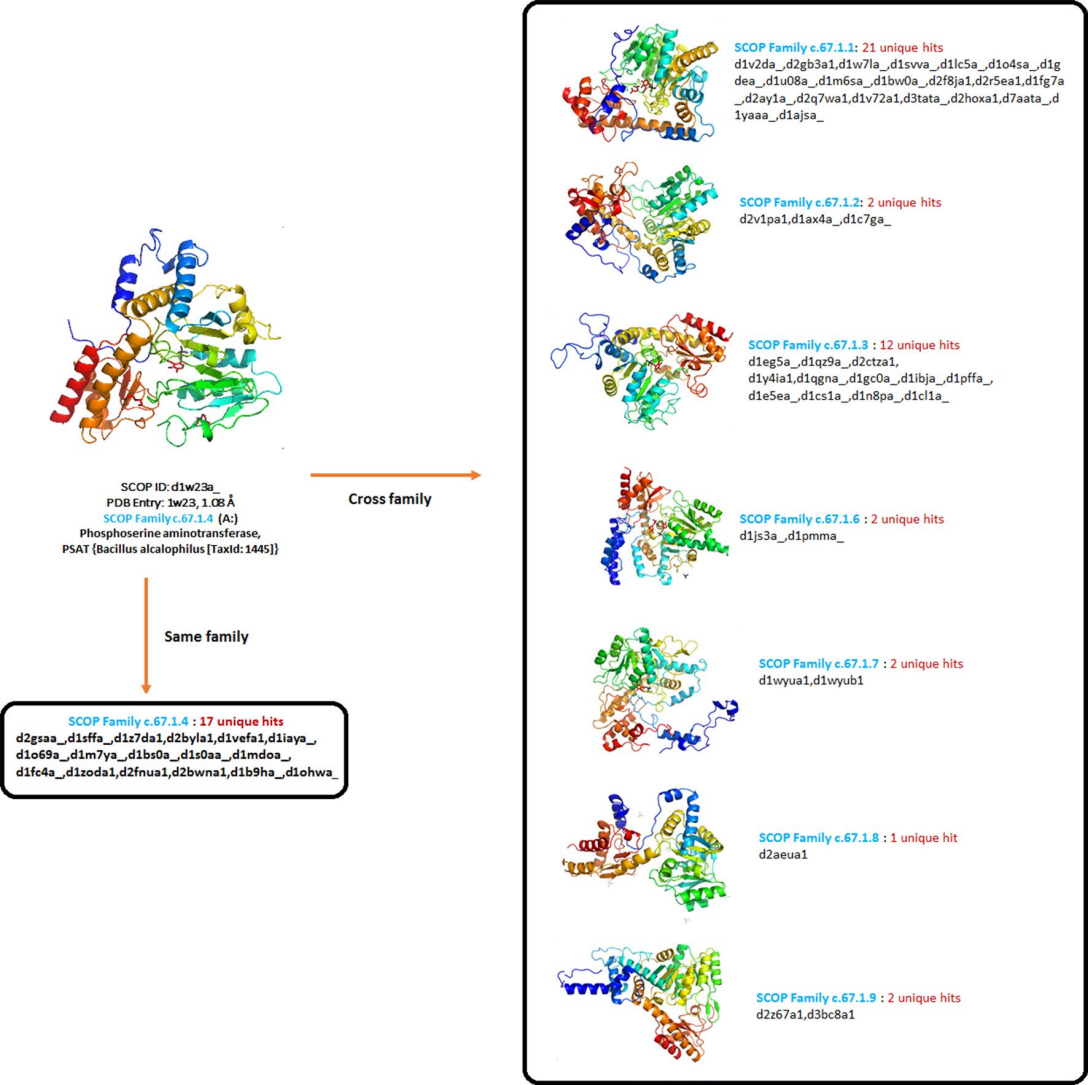
Three-dimensional structure of Phosphoserine aminotransferase, PSAT encoded by *Bacillus alcalophilus* [TaxId: 1445]} (SCOP ID: d1w23a_). Unique hits reported by Master Blaster from the same family and from the cross families are given.

Though the overall sensitivity using HHblits is high compared to Master Blaster, there are many queries for which Master Blaster gave better performance i.e., the hits reported by Master Blaster and that are missed by HHblits. Out of 135,025 true positives reported by Master Blaster, 10,849 are the unique hits reported by Master Blaster using e-value criteria of 1e−2. The 10,849 hits are reported from 46 different folds and these are given in Table 4. These missed hits are not reported by HHblits executed using most optimal parameters. Out of 10,849 unique hits, 9572 are hits from cross-family i.e., homologs from two different families within the same superfamily within a fold, 285 are cross superfamily, and 992 are from the same family. This indicates that Master Blaster performs better in picking remote homologs within the same superfamily. Even using a stringent e-value criterion of 1e−3, there are 8214 unique hits using Master Blaster compared to HHblits. These hits are from 46 different folds (Table 4) and majorly from Immunoglobulin-like beta-sandwich (b.1), Lipocalins (b.60), TIM beta/alpha-barrel (c.1), FAD/NAD(P)-binding domain (c.3), Flavodoxin-like (c.23), PLP-dependent transferase-like (c.67), Thioredoxin fold (c.47), PLP-dependent transferase-like (c.67), and HAD-like (c.108). One example of such unique hits are the remote homologues detected for the members from fold PLP-dependent transferase-like (c.67) i.e., fold having 3 layers of a/b/a, mixed beta-sheet of 7 strands. 57 queries from this particular fold reported unique hits. The unique hits for protein Phosphoserine aminotransferase, PSAT {*Bacillus alcalophilus* [TaxId: 1445]} belonging to this fold PLP-dependent transferase-like (c.67) SCOP ID: d1w23a) is given in Fig. 5. There are few cross superfamily connections, reported using Master Blaster in either second or third generation but not reported using any of the HHblits runs. One such example is the homology between domains from Ribulose-phoshate binding barrel (c.1.2) and Bacterial luciferase-like (c.1.16). The sequence identity between two domains is less than 10%.

**Table 4:** Number of unique hits reported by Master Blaster compared to HHblits.

S. no.	SCOP fold	Number of unique hits
1	a.118	12
2	a.138	12
3	a.1	1
4	a.217	2
5	a.25	14
6	a.39	65
7	a.3	100
8	a.45	8
9	a.4	5
10	b.1	4930
11	b.34	21
12	b.36	6
13	b.45	6
14	b.47	199
15	b.55	19
16	b.60	191
17	b.69	6
18	b.6	1
19	b.81	4
20	b.82	22
21	b.92	86
22	c.108	238
23	c.10	47
24	c.1	69
25	c.23	2127
26	c.2	4
27	c.37	91
28	c.3	559
29	c.46	6
30	c.47	888
31	c.66	73
32	c.67	514
33	c.68	4
34	c.69	114
35	c.79	3
36	c.94	3
37	d.104	9
38	d.108	100
39	d.142	1
40	d.15	13
41	d.17	68
42	d.211	12
43	d.32	13
44	d.38	147
45	d.54	7
46	d.58	29

**Figure 6:**
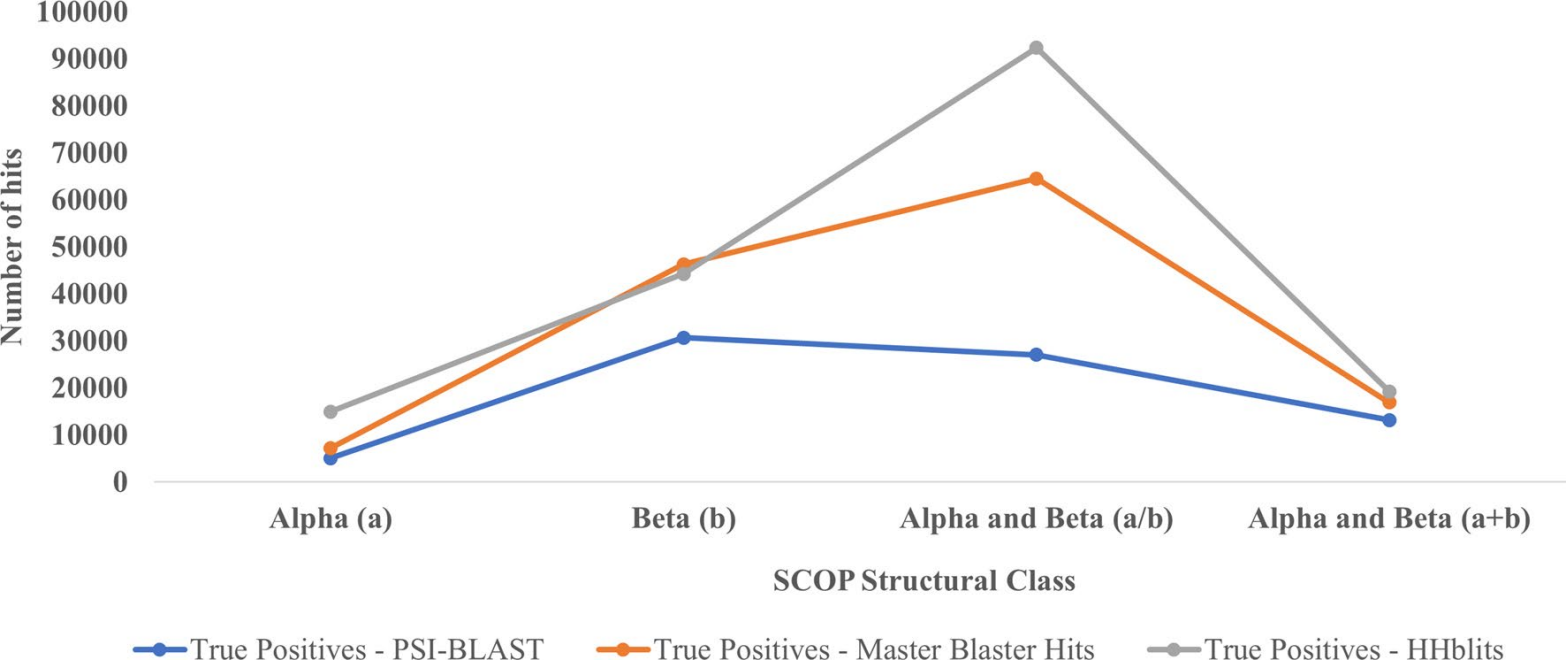
Comparative performance of PSI-BLAST, Master Blaster and HHblits for different structural classes Alpha (a), Beta (b), Alpha and Beta (a/b) and Alpha and Beta (a + b).

An interesting observation with respect to performance of Master Blaster is that it gave better results for beta structural class of protein as can be seen from Table 5. For beta class, Master Blaster reported 46,308 true positives where as HHblits reported 44,282 hits (Fig. 6). Master Blaster reported 117 true positives (47 hits in the first round, 67 hits in the second round and 3 hits in the third round) for the above query sequence (Fig. 5) whereas HHblits reported only 25 true positives even using the best parameters. This clearly signifies the importance of using multiple methods for remote homology detection. Nearly 50% of these unique hits are from the Beta structural class of SCOP.

**Table 5:** Number of domains considered as queries from different structural classes of SCOP and the number of true positives reported using PSI-BLAST, Master Blaster and HHblits.

SCOP class	Number of domains (query sequences)	True positives—PSI-BLAST	True positives—Master Blaster	True positives—HHblits
Alpha (a)	209	5079	7259	14,952
Beta (b)	504	30,677	46,308	44,282
Alpha and Beta (a/b)	865	27,079	64,532	92,345
Alpha and Beta (a + b)	452	13,201	16,926	19,246

### False positives (across fold connections) reported using Master Blaster and HHblits

**Figure 7:**
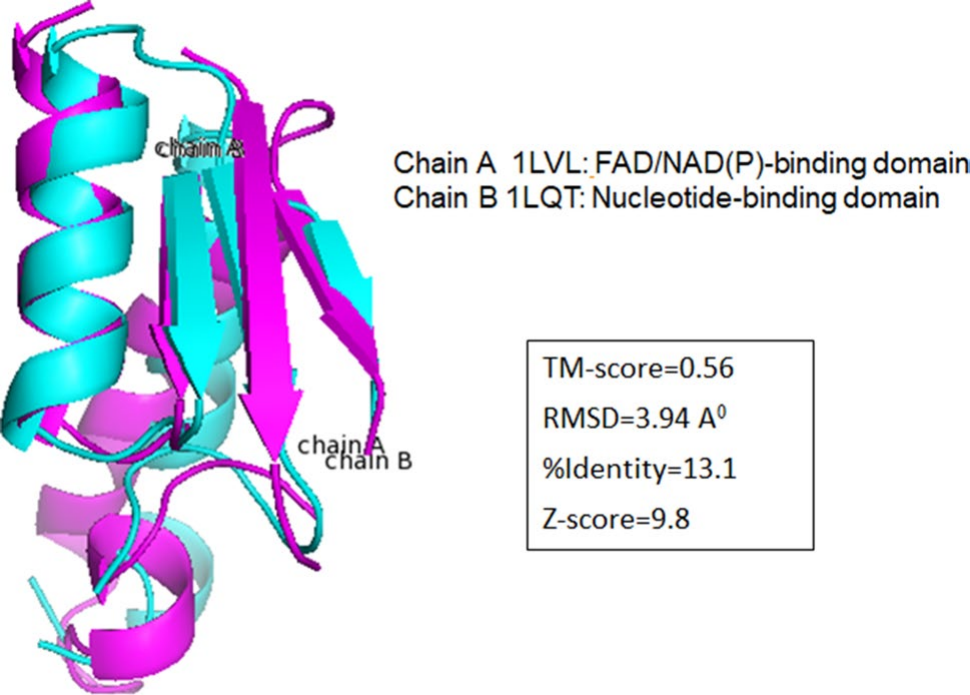
Alignment of three-dimensional structures of local regions of the domains from two different folds FAD/NAD(P)-binding domain (Chain A: PDB code 1LVL) and Nucleotide-binding domain (Chain B: PDB code 1LQT) using TM-Align.

False positives rate is assessed for all the three methods by considering hits from different folds or classes as a false positive. Using E-value cutoff of 1e−2, Master Blaster reported 12,862 false positives and using E-value cutoff of 10^−3^ only 4193 false positives are reported. From the Master Blaster runs, across fold connections are reported from 37 different folds and these false positives are given in Supplementary Table 5. It has been observed that there are very few false positives reported from folds belonging to different classes and 91% of across class connections observed are between protein domains belonging to alpha and beta (a + b) SCOP structural class, and few are reported from beta proteins (b) class to other classes. Majority of across fold connections are reported by queries belonging to Immunoglobulin-like beta-sandwich (b.1), NAD(P)-binding Rossmann-fold domains (c.2) and FAD/NAD(P)-binding domain (c.3), Nucleotide-binding domain (c.4), S-adenosyl-l-methionine-dependent methyltransferases (c.66). Alignment of three-dimensional structures of domains from c.2 and c.3 folds is done using TM-align^35^. Chain A of FAD/NAD(P)-binding domain (PDB Code: 1LVL, SCOP fold: c.3) is aligned to Chain B of Nucleotide-binding domain (PDB Code: 1LQT, SCOP fold: c.4) using TM-align. Interestingly, a significant TM-score (template modeling score) of more than 0.5 is observed in the local regions (Fig. 7). In one of the earlier studies, structural similarity was reported in folds such as Rossman-like folds and the four-to eight bladed β-propellers (c.2–c.5, c.27 and 28, c.30 and 31, b.66–b.70)^36^. Evidence of homology was found between proteins of two ancient and highly populated protein folds^37^, flavodoxin-like fold and the (βα)8-barrel fold. They observed the evolutionary route by which (βα)8-barrel fold is converted to flavodoxin-like in evolution. Maximum sequence identity and structural similarity was found to be confined to the region where the nucleotide binds in proteins belonging to both. It is possible that the shorter (αβ)2 element duplicated and fused multiple times to generate the full (βα)8-barrel fold architecture, whereas the flavodoxin-like fold may have arisen from this with modifications or a duplicated element. From such studies, it is clear that across-fold connections between few folds could be possible because of common evolutionary origin.

HHblits reported 37,035 false positives using the best optimal parameter values i.e., MACT value 0.9 and E-value cutoff of 1e−10. False positive rate for many folds is found to be nearly ten times higher in comparison to Master Blaster.

### Computational time required for the searches

Computational time required for search using various methods are compared (Table 6). All the computations are performed on the super computing resources available at C-DAC Bangalore and at CDAC Pune (http://www.cdac.in). PARAM Yuva-II is a resource part of national PARAM Supercomputing facility (NPSF) at CDAC Pune and has Intel Xeon E5 2670 processors with 16 cores each. As seen in Table 6, the CPU time taken for one iteration of Master Blaster is ten-fold higher than one iteration of HHblits and many folds higher than one iteration of PSI-BLAST. This is because, single iteration of Master Blaster includes multiple steps: one BlastClust run to cluster the sequences having more than a specified %identity and overlap region, one run of multiple sequence alignment using ClustalW to align the intermediate sequence hits, two PSI-BLAST runs: one to generate the PSSM files from ClustalW output using each sequence in the alignment as a master index and the second run is to search the database using each sequence hit as a query in the PSSM format, In the future, the Master Blaster program will be parallelized to exploit the benefit of multicores on the high performance computers.

**Table 6:** CPU time taken to search sequence having average length of 200 amino acids against SCOP70 database having 13,650 domains with an E-value of 0.001 and query coverage of 70%.

Time taken in seconds	PSI-BLAST	Master Blaster	HHblits
CPU time for Single iteration	0m0.079 s	0m32.828 s	0m2.731 s

The computations are performed at CDAC Bangalore on the supercomputing facility having Linux 64-bit platform.

## Conclusions

Sequence-profile and profile-profile based matching methods are known to be effective in detecting distant relationships compared to simple sequence comparison procedures. Iterative database searches using intermediately related sequences are also shown to be effective in recognizing remote homologues. In this study, we developed a new homology detection protocol which combines the power of multiple profiles-based approach and cascade search approach. This protocol named Master Blaster has been assessed by using the sequences of known three dimensional structures and known evolutionary relationships obtained from SCOP database where there are many pairs of related proteins enlisted with very low sequence similarity. Coverage of members at fold, superfamily and family level are analyzed and it has been observed that the method is found to be highly sensitive in detecting remote homologues. Significant improvement in detecting cross superfamily connections in a fold and cross family connections in a superfamily was observed using the new method compared to PSI-BLAST. Overall, an improvement of 50% was observed using Master Blaster. As multiple PSSMs generated using each sequence from multiple sequence alignment are used as queries in every round of Master Blaster, there is a possibility of false positives getting added. These can be minimized by using stringent cutoff for expectation and inclusion threshold values, query coverage and BLASTClust parameters. Performance comparison against hidden Markov based method HHblits showed that HHblits has sensitivity in detecting remote homologues, but there are 10,849 true homologs from 46 SCOP folds that are missed by HHblits and are reported using Master Blaster. There are many across fold and across class hits reported using HHblits indicating a higher error rate compared to Master Blaster. With respect to precision, PSI-BLAST is seen to be more effective in picking the hits with low false positive rate but at the cost of sensitivity whereas HHblits is seen to be more sensitive in picking the remote homologues but at the cost of precision. Master Blaster is seen to be moderate both with respect to sensitivity, precision, and error rate. These observations suggest that it is better to use multiple remote homology detection approaches working on different principles to improve the effectiveness of remote homology detection compared to use of any one method.

This new approach can be applied to large-scale genome analysis where newly sequenced genomes can be functionally annotated by searching against non-redundant sequence databases.

## Materials and methods

### Master Blaster protocol

The steps followed in Master Blaster protocol is depicted in Fig. 1. A query sequence is searched against the sequence database using PSI-BLAST^13^ to start with but executed for multiple iterations. The hits meeting the criteria of query coverage, E and H-value cut-offs are considered for the multiple sequence alignment and generation of PSSM. After performing analysis on the influence of these parameters on remote homology detection, an optimal performance was obtained using an E-value and H-value criteria of 1e−3, number of iterations as 5 and the Query length coverage as 70%. BLASTClust^38^ is used to cluster the hits within a given sequence identity (70%) and the sequence overlap. ClustalW^39^ is used to build multiple sequence alignment for the clustered hits. In the next step, PSI-BLAST is used to generate multiple PSSMs using each sequence in the multiple sequence alignment as a reference. Thus, for ‘n’ hits in the second round after clustering, there will be ‘n’ PSSMs generated and this is similar to the MulPSSM approach. Each PSSM generated in this round is used as a query sequence against the sequence database using PSI-BLAST for multiple iterations.

The homologues qualifying the criteria of query coverage cut-off, E-value and H-value cut-offs are considered for the next round. The cycle of operations of Master Blaster runs using intermediate sequences as queries in the form of PSSM profiles continues until convergence or until number of iterations set is met. ClustalW^39^ is used for multiple sequence alignment of the hits generated in every iteration.

### Datasets used

The method has been assessed using protein domain sequences from Astral representation of SCOP database (http://scop.berkeley.edu/astral/), which consists of relationships among protein domains of known three-dimensional structures^32^. SCOP classifies proteins hierarchically by grouping domains with high sequence similarity into families, families having structural and functional relationships into superfamilies, domains having similar 3-D topology into folds, and domains having a similar arrangement of secondary structural elements into classes^40^. A dataset consisting of 2030 SCOP domains from α-class, β-class, α/β and α + β class are considered and these belong to 153 folds. We considered sequences from only these four structural classes as they are well represented. To assess the performance of Master Blaster against HHblits, ASTRAL SCOP70 (sequences filtered to 70% maximum sequence identity) database having 13,650 SCOP domains is considered. As HHBlits requires a pre-filtered database in hmm format, the database available at http://wwwuser.gwdg.de/~compbiol/data/hhsuite/databases/hhsuite_dbs/ is used. The steps to build the SCOP hmm database for HHblits runs are given by Remmert et al^19^. For Master Blaster runs, the sequences of SCOP domains used to build the SCOP70 database are retrieved using their SCOP identifiers from Astral compendium (http://scop.berkeley.edu/astral/). The query sequences for both Master Blaster and HHblits runs are given in the FASTA format. HHblits internally converts the query sequence into HMM format.

### Metrics used for performance comparison

Each sequence from the above dataset is used as a query against the 13,650 sequences database using Master Blaster, PSI-BLAST and HHblits.

The following four performance measures have been used for evaluation of methods:

$$\% {\text{ Sensitivity or Recall }} = {\text{ True Positive Rate }}\left( {{\text{TPR}}} \right) \, = \, \left( {{\text{TP}}/\left( {{\text{TP}} + {\text{FN}}} \right)} \right)$$

$$\% {\text{ Precision or Positive predictive value }} = \, \left( {{\text{TP}}/\left( {{\text{TP}} + {\text{FP}}} \right)} \right)$$

$$\% {\text{ Specificity }} = \, \left( {{\text{TN}}/\left( {{\text{TN}} + {\text{FP}}} \right)} \right)$$

$$\% {\text{ Error rate }} = \, \left( {{\text{FP}}/\left( {{\text{FP}} + {\text{TP}}} \right)} \right)$$

If the domain which is reported as a hit belong to the same fold as that of query, it is considered as true positive (TP). False negatives (FN) are the domains belonging to the same fold as that of the query but could not be detected as a hit. False positives (FP) are the hits that belong to a different fold as that of the query. True negatives (TN) are the sequences belonging to a different fold as that of query sequence. For example, the number of true negatives for the fold Immunoglobulin-like beta-sandwich (b.1) containing 402 members in the SCOP70 dataset containing 13,650 sequences is 13,248 (13,650-402). For the Master Blaster runs, the aligned regions of intermediate sequences or hits from each generation that qualify a specific E-value and H-value cut-off and query coverage cut-off are considered for analysis. As the length of sequence alignment using intermediate sequence hits can shorten with every subsequent round, hits that has an overlap of 70% or above with the original query sequence are generally considered to be significant. Length criteria can help to avoid false positives that arise due to short alignments.

## Supplementary Information

Supplementary Information.

Supplementary Table S1.

Supplementary Table S2.

Supplementary Table S3.

Supplementary Table S4.

Supplementary Table S5.

## Data Availability

All the data used in this work are available in publicly available databases as described in the paper. Data derived from this work are available in supplementary information.

## Code availability

Master Blaster stand-alone code is available for download in the supplementary archive.
