# Supplementary material for: Master Blaster: an approach to sensitive identification of remotely related proteins
Source: Sci Rep. 2021 Apr 22;11:8746. doi: 10.1038/s41598-021-87833-4 (PMC8062480; doi:10.1038/s41598-021-87833-4)
Supplement: Supplementary file 1 — Supplementary Information. [file 41598_2021_87833_MOESM1_ESM.pdf]

```
#Ch. Janaki - Master BLaster Wrapper Code
#
#Readme file - instructions to Run Master Blaster Wrapper Code. Master
Blaster makes use of PSI-BLAST, ClustalW, BlastClust and some pre & post
processing scripts #
-----------------------------------------------------------------------
#Packages/Scripts to be Downloaded before execution of Master Blaster

#faSomeRecords - https://github.com/santiagosnchez/faSomeRecords
#blast-2.2.28+ -
#ftp://ftp.ncbi.nlm.nih.gov/blast/executables/blast+/2.2.28/
#Clustalw-2.0.12 - http://www.clustal.org/download/2.0.12/
#blastclust - Available with NCBI BLAST Package. Blastclust part of
#blast-2.2.19 is used in this wrapper code.
#Splitfasta.pl - https://github.com/josephhughes/Sequence-
manipulation/blob/master/SplitFasta.pl
#processfasta.pl - Needs Bioperl installation.
-----------------------------------------------------------------------

#The input files in fasta format (.fsa) to be made available in the
directory where the Master Blaster code runs. An output directory will be
created with inputfilename.dir
#
#For example, if test.fsa is given as input, output folder will be
test.fsa.dir
#
#Database need to be in fasta format. Else the job will not run. Example
~/MB/scop70_1.75_ids.seq  is the sequence database in fasta format
#To run Master Blaster wrapper code – Save the below wrapper script as
MasterBlaster_Wrapper_Code.sh
#
#sh MasterBlaster_Wrapper_Code.sh

#!/bin/sh
#source /opt/app/BioPerl-1.6.922/env.sh
#MASTER BLASTER
#Read file in Fasta format
for file in *.fsa
do
mkdir "$file.dir"
cp $file $file.dir
currdir=$(pwd)
dir=$currdir/$file.dir

#STEP to get the length of Query sequence file
      length=$(awk '$0 ~ ">" {print c; c=0;printf substr($0,2,100) "\t";
} $0 !~ ">" {c+=length($0);} END { print c; }' $file|awk '{print $NF}')

#STEP to get the Query ID

      queryid=$(awk '$0 ~ ">" {print c; c=0;printf substr($0,2,100) "\t";
} $0 !~ ">" {c+=length($0);} END { print c; }' $file|awk '{print $1}')

#STEP to calculate 70% Query sequence length

value_length=`echo "scale=1; $length * 0.6 " | bc`
echo "print statement 1 $value_length"
```

```
#STEP to Execute Psiblast - 1st run. blast-2.2.28+ version is used.

      ~/blast-2.2.28+/psiblast -query $file -db ~/MB/scop70_1.75_ids.seq
-num_threads 4 -evalue 0.001 -out $dir/$file.psiblastout.1  -outfmt '10
qseqid qlen sseqid length evalue qstart qend sstart send pident' -
inclusion_ethresh 0.001 -num_iterations 5

      sed -i "s/,/\ /g" $dir/$file.psiblastout.1

#STEP to get 70% overlap hits from PSIBLAST OUTPU
T
      awk -v len="$value_length" '{if ($4>=len) print}'
$dir/$file.psiblastout.1 > $dir/$file.psiblastout.1.70
      awk '{print $3}' $dir/$file.psiblastout.1.70 |grep -v
'CONVERGED'|awk '!x[$0]++' > $dir/$file.allhitspssm.1.accno


#Step to retrieve sequences based on Accesion Ids and Positions
########################################################################
################################################
#faSomeRecords to be downloaded. Details given in README file
#Splitfasta.pl to be downloaded. Details given in README file

      ~/MB/faSomeRecords ~/MB/scop70_1.75_ids.seq
$dir/$file.allhitspssm.1.accno $dir/$file.allhitspssm.1.accno.seq
        perl ~/Splitfasta.pl --input_file
$dir/$file.allhitspssm.1.accno.seq --output_dir $dir/
      for fileaccno in $dir/$file.allhitspssm.1.accno
      do
            count=0
            cat $fileaccno | while read SCOPID
            do
                    let count++
            echo $SCOPID > $dir/$SCOPID.ID
#faSomeRecords is a script to fetch sequences based on unique IDs. Two
input files to be given: 1) File having unique ids 2)File having all
sequences (database)

            awk -v line="$SCOPID" '{if ($3==line){print $3 " " $4 " " $8
" " $9}}' $dir/$file.psiblastout.1.70|awk '!x[$0]++' >
$dir/$SCOPID.pos.temp
            value=$(awk 'BEGIN {max = 0} {if ($2>max) max=$2} END {print
max}' $dir/$SCOPID.pos.temp)
            grep -m 1 $value $dir/$SCOPID.pos.temp > $dir/$SCOPID.pos

             sstart=$(awk '{print $3}' $dir/$SCOPID.pos)
             send=$(awk '{print $4}' $dir/$SCOPID.pos)
             /usr/bin/perl ~/process_fasta.pl $dir/$SCOPID.seq $sstart
$send

            cat $dir/$SCOPID.seq.out >>
$dir/$file.run.1.commonseq_beforeclust
            done
      done

# Step to remove redundant sequences based on a particular cutoff. -S
option is   parameter value passed to BLASTClust to set % sequence
similarity and -L is used for length coverage
```

```
      ~/blast-2.2.19/bin/blastclust -i
$dir/$file.run.1.commonseq_beforeclust -o
$dir/$file.run.1.commonseq_clust  -S 90 -L .7

      awk '{print $1}' $dir/$file.run.1.commonseq_clust >
$dir/$file.run.1.commonseq_clustid

      ~/MB/faSomeRecords $dir/$file.run.1.commonseq_beforeclust
$dir/$file.run.1.commonseq_clustid $dir/$file.run.1.commonseq


#Step to Execute Clustalw on output generated by PSI-BLAST in earlier
step

      clustalw2 -INFILE=$dir/$file.run.1.commonseq -
OUTFILE=$dir/$file.run.1.align -QUIET

      tail -n +4 $dir/$file.run.1.align > $dir/$file.run.1.alignment

#STEP to execute PSI-BLAST using  Clustalw alignment output using all the
hits as references automatically by incrementing msa_master_idx value

      for ((iter=1; iter<=5 ; iter++))
      do
        length=$(awk '$0 ~ ">" {print c; c=0;printf substr($0,2,100)
"\t"; } $0 !~ ">" {c+=length($0);} END { print c; }' $file|awk '{print
$NF}')
        value_length=`echo "scale=1; $length * 0.6 " | bc`
echo "print statement 1 in for loop $value_length"
      iter1=$(($iter+1))
      lineno=$(sed -e '/^$/,$d' $dir/$file.run.$iter.alignment|wc -l)
      nolines=$(( $lineno - 1 ))
            for ((i=1; i<=$nolines; i++ )) ;
            do
                  queryid2=$( awk 'NR=='$i' {print;exit}'
$dir/$file.run.$iter.alignment|awk '{print $1}')
      ~/blast-2.2.28+/psiblast -in_msa $dir/$file.run.$iter.alignment -db
~/MB/scop70_1.75_ids.seq -num_threads 4 -evalue 0.001 -out_pssm
$dir/$file.$i.pssm.$iter1  -out $dir/$file.$i.psiblastmsa.$iter1 -outfmt
'10 qseqid qlen sseqid length evalue pident' -num_iterations 3 -
msa_master_idx $i -inclusion_ethresh 0.001
      sed -i -e "s/unnamed/"$queryid2"/g" $dir/$file.$i.pssm.$iter1
      done

##########################################################################
##################3333333333

# In every iteration, PSIBLAST uses  different sequences as queries. This
Code to get Queryis dof each run

            k=0;
            for j in $dir/*.pssm.$iter1
            do
                  k=$(( $k + 1 ))
                  querystring=$(grep  "local str" $j|awk '{print $NF}'|sed
-e 's/"//g')
                  ~/blast-2.2.28+/psiblast -in_pssm $j -db
~/MB/scop70_1.75_ids.seq -num_threads 4 -evalue 0.001 -out
```

```
$dir/$file.$k.psiblastpssm.$iter1  -outfmt '10 qseqid qlen sseqid length
evalue  qstart qend sstart send  pident' -num_iterations 3 -
inclusion_ethresh 0.001
                sed -i -e "s/unnamed/"$querystring"/g"
$dir/$file.$k.psiblastpssm.$iter1
                cat $dir/$file.*.psiblastpssm.$iter1 >
$dir/$file.allhitspssm.run.$iter1
                sed -i "s/,/\ /g" $dir/$file.allhitspssm.run.$iter1
                         echo "print statement 2 $value_length"
                awk -v len="$value_length" '{if
(($4>=len)&&($NF<100)){print}}' $dir/$file.allhitspssm.run.$iter1 >
$dir/$file.allhits.psiblastpssm.$iter1.70
                awk '{print $3}'
$dir/$file.allhits.psiblastpssm.$iter1.70 |grep -v 'CONVERGED'|awk
'!x[$0]++'|sed '/^$/d' > $dir/$file.allhitspssm.$iter1.accno
                line_no3=$(awk '{x++} END {print x}'
$dir/$file.allhitspssm.$iter1.accno)
          done

#Step to compare results of run2 and run3. If results converge, the job
will stop. Else will continue for next iteration

          /usr/bin/perl ~/MB/scriptunique
$dir/$file.allhitspssm.$iter.accno $dir/$file.allhitspssm.$iter1.accno >
$dir/$file.unique_run.$iter1
          for ((iter2=1; iter2<=$iter; iter2++ ));
          do
                cat $dir/$file.allhitspssm.$iter2.accno|awk -v
query="$queryid" '{if ($1!=query){print}}' >>
$dir/$file.allhitspssm.$iter.accno_all
          done
          awk -v query="$queryid" '{if ($1!=query){print}}'
$dir/$file.allhitspssm.$iter1.accno >
$dir/$file.allhitspssm.$iter1.accno_noquery
          /usr/bin/perl ~/MB/scriptunique
$dir/$file.allhitspssm.$iter.accno_all
$dir/$file.allhitspssm.$iter1.accno_noquery >
$dir/$file.unique_run_all.$iter1

          line_no4=$(awk '{x++} END {print x}'
$dir/$file.unique_run_all.$iter1)


#STEP to run clustalw after checking the condition "If there are unique
hits in run3"
          if [ "$line_no4" -gt 0 ]
          then
#Step to retrieve sequences based on Accesion Ids and Positions
          for fileaccno in $dir/$file.allhitspssm.$iter1.accno
          do
                count=0
                cat $fileaccno | while read SCOPID
                do
                let count++
                  echo $SCOPID > $dir/$SCOPID.ID
                ~/MB/faSomeRecords ~/MB/scop70_1.75_ids.seq
$dir/$SCOPID.ID $dir/$SCOPID.seq
```

```
                  awk -v line="$SCOPID" '{if ($3==line){print $3 " " $4 "
" $8 " " $9}}' $dir/$file.allhits.psiblastpssm.$iter1.70|awk '!x[$0]++' >
$dir/$SCOPID.pos.temp
                    value2=$(awk 'BEGIN {max = 0} {if ($2>max) max=$2} END
{print max}' $dir/$SCOPID.pos.temp)
                     grep -m 1 $value2 $dir/$SCOPID.pos.temp >
$dir/$SCOPID.pos
              #            echo "print statement 5 $value"
                   sstart2=$(awk '{print $3}' $dir/$SCOPID.pos)
                   send2=$(awk '{print $4}' $dir/$SCOPID.pos)
                   /usr/bin/perl ~/process_fasta.pl $dir/$SCOPID.seq
$sstart2 $send2
                  cat $dir/$SCOPID.seq.out >>
$dir/$file.$iter1.commonseq_beforeclust
                  done
            done
#Second run of Blastclust
            ~/blast-2.2.19/bin/blastclust -i
$dir/$file.$iter1.commonseq_beforeclust -o
$dir/$file.$iter1.commonseq_clust -S 80 -L .7
            awk '{print $1}' $dir/$file.$iter1.commonseq_clust >
$dir/$file.$iter1.commonseq_clustid
            ~/MB/faSomeRecords $dir/$file.$iter1.commonseq_beforeclust
$dir/$file.$iter1.commonseq_clustid $dir/$file.$iter1.commonseq

#Step to Execute Clustalw

            clustalw2 -QUIET -INFILE=$dir/$file.$iter1.commonseq -
OUTFILE=$dir/$file.run.$iter1.align

#Step to prepare the alignment file by trimming first three lines of
$file.align

            tail -n +4 $dir/$file.run.$iter1.align >
$dir/$file.run.$iter1.alignment

      else
            echo "Results converged"
      break
      fi
      done
done
#remove temporary files
\rm $dir/*temp*
\rm $dir/*.psiblastpssm.*
\rm $dir/*.psiblastmsa.*
\rm $dir/*.pssm.*
\rm $dir/*.ID
\rm $dir/*.seq.*
\rm $dir/*.seq
\rm $dir/*.pos
```
