# Supplementary material for: Master Blaster: an approach to sensitive identification of remotely related proteins
Source: Sci Rep. 2021 Apr 22;11:8746. doi: 10.1038/s41598-021-87833-4 (PMC8062480; doi:10.1038/s41598-021-87833-4)
Supplement: Supplementary file 2 — Supplementary Table S1. [file 41598_2021_87833_MOESM2_ESM.docx]

**Title: Master Blaster: An approach to sensitive identification of remotely related proteins**

Authors: Chintalapati Janaki, Venkatasubramanian S. Gowri and Narayanaswamy Srinivasan

**Supplementary table 1**: Influence of E-value, H-value, and Query Coverage on Master Blaster performance.

| TP – True Positives, FP- False Positives, FN – False Negatives, TN – True Negatives |  | |  | |
| --- | --- | --- | --- | --- |
| % Sensitivity or Recall = True Positive Rate (TPR) = (TP/(TP+FN))  % Precision or Positive predictive value = (TP/(TP+FP))  % Specificity = (TN/(TN+FP))  % Error rate = (FP/(FP+TP))   \| **E-value & H-value** \| **Query coverage** \| **Precision** \| **Error Rate** \| **Sensitivity/TPR** \| **Specificity** \| \| --- \| --- \| --- \| --- \| --- \| --- \| \| 1e-3 \| 60% \| 0.90 \| 0.10 \| 0.34 \| 0.99 \| \| 1e-3 \| 70% \| 0.97 \| 0.03 \| 0.32 \| 1.00 \| \| 1e-2 \| 60% \| 0.89 \| 0.11 \| 0.38 \| 0.99 \| \| 1e-2 \| 70% \| 0.91 \| 0.09 \| 0.36 \| 0.99 \| |  |  | |  |
|  |  | |  | |
|  |  | |  | |
