# Supplementary material for: Master Blaster: an approach to sensitive identification of remotely related proteins
Source: Sci Rep. 2021 Apr 22;11:8746. doi: 10.1038/s41598-021-87833-4 (PMC8062480; doi:10.1038/s41598-021-87833-4)
Supplement: Supplementary file 3 — Supplementary Table S2. [file 41598_2021_87833_MOESM3_ESM.docx]

**Title: Master Blaster: An approach to sensitive identification of remotely related proteins**

Authors: Chintalapati Janaki, Venkatasubramanian S. Gowri and Narayanaswamy Srinivasan

**Supplementary table 2**: Influence of Number of iterations, E-value, Mact value and %Query Coverage on HHBlits performance.

TP – True Positives, FP- False Positives, FN – False Negatives, TN – True Negatives

% Sensitivity or Recall = True Positive Rate (TPR) = (TP/(TP+FN))

% Precision or Positive predictive value = (TP/(TP+FP))

% Specificity = (TN/(TN+FP))

% Error rate = (FP/(FP+TP))

| **S.No** | **Number of iterations** | **E-Value** | **Mact Value** | **%Query coverage** | **Precision** | **Error Rate** | **Sensitivity/TPR** | **Specificity** |
| --- | --- | --- | --- | --- | --- | --- | --- | --- |
|  | 1 | 10 | 0.6 | 70 | 0.79 | 0.2 | 0.4 | .98 |
|  | 1 | 10 | 0.9 | 70 | 0.82 | 0.17 | 0.39 | .98 |
|  | 1 | 2 | 0.35 | 70 | 0.76 | 0.23 | 0.44 | .97 |
|  | 1 | 3 | 0.35 | 70 | 0.76 | 0.23 | 0.44 | .97 |
|  | 1 | 4 | 0.35 | 70 | 0.76 | 0.23 | 0.45 | .97 |
|  | 1 | 5 | 0.35 | 70 | 0.76 | 0.23 | 0.45 | .97 |
|  | 1 | 6 | 0.35 | 70 | 0.76 | 0.23 | 0.45 | .97 |
|  | 1 | 7 | 0.35 | 70 | 0.76 | 0.23 | 0.45 | .97 |
|  | 1 | 8 | 0.35 | 70 | 0.76 | 0.23 | 0.46 | .97 |
|  | 1 | 9 | 0.35 | 70 | 0.76 | 0.23 | 0.46 | .97 |
|  | 2 | 10 | 0.9 | 70 | 0.82 | 0.17 | 0.45 | .98 |
|  | 2 | 2 | 0.35 | 70 | 0.76 | 0.23 | 0.44 | .97 |
|  | 2 | 3 | 0.35 | 70 | 0.76 | 0.23 | 0.44 | .97 |
|  | 2 | 4 | 0.35 | 70 | 0.76 | 0.23 | 0.45 | .97 |
|  | 2 | 5 | 0.35 | 70 | 0.76 | 0.23 | 0.45 | .97 |
|  | 2 | 6 | 0.35 | 70 | 0.76 | 0.23 | 0.45 | .97 |
|  | 2 | 7 | 0.35 | 70 | 0.76 | 0.23 | 0.45 | .97 |
|  | 2 | 8 | 0.35 | 70 | 0.76 | 0.23 | 0.46 | .97 |
|  | 2 | 9 | 0.35 | 70 | 0.76 | 0.23 | 0.46 | .97 |
|  | 3 | 10 | 0.6 | 60 | 0.8 | 0.19 | 0.46 | .98 |
|  | 3 | 10 | 0.6 | 70 | 0.8 | 0.19 | 0.47 | .97 |
|  | 3 | 10 | 0.9 | 70 | 0.82 | 0.17 | 0.45 | .98 |
|  | 3 | 2 | 0.35 | 70 | 0.75 | 0.24 | 0.44 | .97 |
|  | 3 | 3 | 0.35 | 70 | 0.75 | 0.24 | 0.44 | .97 |
|  | 3 | 3 | 0.6 | 70 | 0.8 | 0.19 | 0.46 | .97 |
|  | 3 | 3 | 0.6 | 80 | 0.78 | 0.21 | 0.47 | .97 |
|  | 3 | 4 | 0.35 | 70 | 0.76 | 0.23 | 0.45 | .97 |
|  | 3 | 5 | 0.35 | 70 | 0.75 | 0.24 | 0.45 | .97 |
|  | 3 | 6 | 0.35 | 70 | 0.76 | 0.23 | 0.45 | .97 |
|  | 3 | 6 | 0.3 | 80 | 0.79 | 0.2 | 0.45 | .97 |
|  | 3 | 6 | 0.4 | 70 | 0.8 | 0.19 | 0.45 | .98 |
|  | 3 | 6 | 0.6 | 80 | 0.78 | 0.21 | 0.47 | .97 |
|  | 3 | 7 | 0.35 | 70 | 0.75 | 0.24 | 0.46 | .97 |
|  | 3 | 8 | 0.35 | 70 | 0.75 | 0.24 | 0.46 | .97 |
|  | 3 | 8 | 0.4 | 70 | 0.8 | 0.19 | 0.45 | .98 |
|  | 3 | 8 | 0.9 | 70 | 0.82 | 0.17 | 0.45 | .98 |
|  | 3 | 9 | 0.35 | 70 | 0.75 | 0.24 | 0.46 | .97 |
|  | 4 | 10 | 0.9 | 70 | 0.82 | 0.17 | 0.45 | .98 |
|  | 4 | 2 | 0.35 | 70 | 0.75 | 0.24 | 0.44 | .97 |
|  | 4 | 3 | 0.35 | 70 | 0.75 | 0.24 | 0.44 | .97 |
|  | 4 | 4 | 0.35 | 70 | 0.76 | 0.23 | 0.45 | .97 |
|  | 4 | 5 | 0.35 | 70 | 0.75 | 0.24 | 0.45 | .97 |
|  | 4 | 6 | 0.35 | 70 | 0.76 | 0.23 | 0.45 | .97 |
|  | 4 | 7 | 0.35 | 70 | 0.75 | 0.24 | 0.46 | .97 |
|  | 4 | 8 | 0.35 | 70 | 0.75 | 0.24 | 0.46 | .97 |
|  | 4 | 9 | 0.35 | 70 | 0.75 | 0.24 | 0.46 | .97 |
|  | 5 | 10 | 0.2 | 70 | 0.79 | 0.2 | 0.45 | .97 |
|  | 5 | 10 | 0.35 | 70 | 0.79 | 0.2 | 0.45 | .97 |
|  | 5 | 10 | 0.3 | 70 | 0.79 | 0.2 | 0.45 | .97 |
|  | 5 | 10 | 0.4 | 70 | 0.8 | 0.19 | 0.45 | .97 |
|  | 5 | 10 | 0.5 | 70 | 0.8 | 0.19 | 0.46 | .98 |
|  | 5 | 10 | 0.6 | 70 | 0.8 | 0.19 | 0.47 | .97 |
|  | 5 | 10 | 0.6 | 80 | 0.78 | 0.21 | 0.47 | .97 |
|  | 5 | 10 | 0.9 | 70 | 0.82 | 0.17 | 0.45 | .98 |
|  | 5 | 10 | 0.9 | 80 | 0.81 | 0.18 | 0.44 | .98 |
|  | 5 | 2 | 0.35 | 70 | 0.75 | 0.24 | 0.44 | .97 |
|  | 5 | 3 | 0.35 | 70 | 0.75 | 0.24 | 0.44 | .97 |
|  | 5 | 3 | 0.6 | 80 | 0.78 | 0.21 | 0.47 | .97 |
|  | 5 | 4 | 0.35 | 70 | 0.76 | 0.23 | 0.45 | .97 |
|  | 5 | 4 | 0.5 | 80 | 0.78 | 0.21 | 0.46 | .97 |
|  | 5 | 5 | 0.35 | 70 | 0.75 | 0.24 | 0.45 | .97 |
|  | 5 | 6 | 0.2 | 70 | 0.79 | 0.2 | 0.44 | .97 |
|  | 5 | 6 | 0.35 | 70 | 0.76 | 0.23 | 0.45 | .97 |
|  | 5 | 6 | 0.3 | 80 | 0.79 | 0.2 | 0.45 | .97 |
|  | 5 | 6 | 0.4 | 70 | 0.8 | 0.19 | 0.45 | .98 |
|  | 5 | 6 | 0.5 | 80 | 0.78 | 0.21 | 0.46 | .97 |
|  | 5 | 7 | 0.35 | 70 | 0.75 | 0.24 | 0.46 | .97 |
|  | 5 | 8 | 0.2 | 80 | 0.78 | 0.21 | 0.45 | .97 |
|  | 5 | 8 | 0.35 | 70 | 0.75 | 0.24 | 0.46 | .97 |
|  | 5 | 8 | 0.6 | 60 | 0.8 | 0.19 | 0.42 | .98 |
|  | 5 | 8 | 0.6 | 70 | 0.8 | 0.19 | 0.46 | .97 |
|  | 5 | 9 | 0.35 | 70 | 0.75 | 0.24 | 0.46 | .97 |
