# Supplementary material for: Master Blaster: an approach to sensitive identification of remotely related proteins
Source: Sci Rep. 2021 Apr 22;11:8746. doi: 10.1038/s41598-021-87833-4 (PMC8062480; doi:10.1038/s41598-021-87833-4)
Supplement: Supplementary file 4 — Supplementary Table S3. [file 41598_2021_87833_MOESM4_ESM.docx]

**Title: Master Blaster: An approach to sensitive identification of remotely related proteins**

Authors: Chintalapati Janaki, Venkatasubramanian S. Gowri and Narayanaswamy Srinivasan

**Supplementary table 3**: Performance comparison of PSI-BLAST and Master Blaster with respect to cross superfamily (CSF), cross family (CF), and same or Intra family (SFAM) connections.

PSI-BLAST versus Master Blaster

TP - True Positives

SFAM – Hits (TPs) from the Same Family or Intra family

CF- Hits (TPs) from the Cross Family,

CSF – Hits (TPs) from the Cross Superfamily

| **Parameters** | **CSF_PSI-BLAST** | **CSF_Master Blaster** | **%Improvement** |
| --- | --- | --- | --- |
| **E-value & H-value - 1e-3, 60% query coverage** | 88 | 980 | 8.98 |
| **E-value & H-value- 1e-3, 70% query coverage** | 59 | 755 | 7.81 |
| **E-value & H-value - 1e-2, 60% query coverage** | 219 | 1790 | 12.23 |
| **E-value & H-value - 1e-2, 70% query coverage** | 164 | 1147 | 14.3 |

| **Parameters** | **CF_PSI-BLAST** | **CF_Master Blaster** | **%Improvement** |
| --- | --- | --- | --- |
| **E-value & H-value - 1e-3, 60% query coverage** | 23006 | 66520 | 34.59 |
| **E-value & H-value- 1e-3, 70% query coverage** | 20780 | 60417 | 34.39 |
| **E-value & H-value - 1e-2, 60% query coverage** | 29083 | 80122 | 36.3 |
| **E-value & H-value - 1e-2, 70% query coverage** | 26025 | 74299 | 35.03 |

| **Parameters** | **SFAM_PSI-BLAST** | **SFAM_Master Blaster** | **%Improvement** |
| --- | --- | --- | --- |
| **E-value & H-value - 1e-3, 60% query coverage** | 49775 | 59117 | 84.2 |
| **E-value & H-value- 1e-3, 70% query coverage** | 47624 | 58173 | 81.87 |
| **E-value & H-value - 1e-2, 60% query coverage** | 52175 | 60507 | 86.23 |
| **E-value & H-value - 1e-2, 70% query coverage** | 49847 | 59579 | 83.67 |
