# Supplementary material for: Master Blaster: an approach to sensitive identification of remotely related proteins
Source: Sci Rep. 2021 Apr 22;11:8746. doi: 10.1038/s41598-021-87833-4 (PMC8062480; doi:10.1038/s41598-021-87833-4)
Supplement: Supplementary file 5 — Supplementary Table S4. [file 41598_2021_87833_MOESM5_ESM.docx]

**Title: Master Blaster: An approach to sensitive identification of remotely related proteins**

Authors: Chintalapati Janaki, Venkatasubramanian S. Gowri and Narayanaswamy Srinivasan

**Supplementary Table 4** – True Positives (TPs) reported by HHblits runs using E-value of 10^-10^, Number of iterations as 5, Query coverage – 70% and with different MACT values i.e., 0.35, 0.5, 0.6, 0.9.

Significant differences in the number of true positives are highlighted in Red.

| **SCOP Fold** | **TP - MACT -0.35** | **TP-MACT -0.5** | **TP- MACT -0.6** | **TP- MACT -0.9** |
| --- | --- | --- | --- | --- |
| a.1 | 569 | 897 | 891 | 858 |
| a.102 | 14 | 12 | 11 | 11 |
| a.104 | 21 | 21 | 21 | 21 |
| a.118 | 118 | 116 | 116 | 110 |
| a.130 | 6 | 7 | 6 | 6 |
| a.132 | 76 | 77 | 77 | 62 |
| a.138 | 47 | 48 | 47 | 37 |
| a.2 | 5 | 5 | 5 | 5 |
| a.21 | 26 | 26 | 26 | 26 |
| a.211 | 14 | 14 | 14 | 14 |
| a.217 | 5 | 5 | 5 | 3 |
| a.22 | 168 | 168 | 167 | 157 |
| a.25 | 609 | 601 | 646 | 723 |
| a.28 | 11 | 11 | 11 | 11 |
| a.3 | 1298 | 1384 | 1437 | 1316 |
| a.35 | 74 | 74 | 74 | 76 |
| a.38 | 8 | 8 | 8 | 8 |
| a.39 | 2532 | 2616 | 2663 | 2626 |
| a.4 | 6992 | 6955 | 7056 | 7784 |
| a.40 | 48 | 48 | 48 | 48 |
| a.45 | 665 | 653 | 675 | 675 |
| a.5 | 256 | 255 | 254 | 248 |
| a.60 | 53 | 51 | 51 | 49 |
| a.7 | 11 | 11 | 11 | 11 |
| a.74 | 51 | 50 | 50 | 47 |
| a.96 | 20 | 20 | 20 | 20 |
| b.1 | 31414 | 32110 | 33707 | 30861 |
| b.113 | 6 | 6 | 6 | 6 |
| b.121 | 96 | 99 | 82 | 79 |
| b.122 | 11 | 12 | 11 | 9 |
| b.21 | 7 | 7 | 7 | 7 |
| b.22 | 18 | 18 | 18 | 16 |
| b.23 | 14 | 14 | 14 | 14 |
| b.26 | 26 | 26 | 26 | 26 |
| b.29 | 84 | 86 | 86 | 81 |
| b.3 | 23 | 22 | 22 | 22 |
| b.33 | 102 | 102 | 102 | 102 |
| b.34 | 1843 | 1848 | 1851 | 1774 |
| b.35 | 395 | 394 | 397 | 396 |
| b.36 | 3446 | 3444 | 3500 | 3493 |
| b.38 | 89 | 88 | 88 | 83 |
| b.40 | 72 | 71 | 72 | 68 |
| b.42 | 134 | 134 | 134 | 119 |
| b.43 | 91 | 90 | 90 | 83 |
| b.45 | 145 | 157 | 155 | 123 |
| b.47 | 2375 | 2382 | 2419 | 2352 |
| b.49 | 16 | 16 | 16 | 16 |
| b.50 | 139 | 134 | 135 | 130 |
| b.52 | 48 | 46 | 46 | 43 |
| b.55 | 1149 | 1248 | 1251 | 1294 |
| b.6 | 1479 | 1426 | 1513 | 1406 |
| b.60 | 147 | 165 | 186 | 209 |
| b.68 | 34 | 34 | 34 | 35 |
| b.69 | 141 | 141 | 141 | 138 |
| b.7 | 186 | 187 | 187 | 187 |
| b.71 | 29 | 34 | 34 | 19 |
| b.72 | 14 | 14 | 14 | 14 |
| b.80 | 19 | 18 | 18 | 18 |
| b.81 | 176 | 172 | 169 | 155 |
| b.82 | 481 | 525 | 514 | 557 |
| b.85 | 40 | 40 | 40 | 40 |
| b.92 | 369 | 363 | 360 | 307 |
| c.1 | 6798 | 6753 | 6808 | 5975 |
| c.10 | 223 | 223 | 226 | 154 |
| c.108 | 3168 | 3214 | 3196 | 3178 |
| c.123 | 4 | 4 | 4 | 4 |
| c.14 | 102 | 100 | 100 | 100 |
| c.2 | 24807 | 25047 | 25470 | 24368 |
| c.23 | 819 | 848 | 907 | 799 |
| c.26 | 314 | 312 | 312 | 306 |
| c.3 | 4090 | 4116 | 4143 | 4117 |
| c.31 | 115 | 115 | 115 | 92 |
| c.33 | 24 | 24 | 24 | 24 |
| c.36 | 38 | 39 | 40 | 40 |
| c.37 | 27787 | 27858 | 27948 | 28753 |
| c.4 | 27 | 27 | 27 | 27 |
| c.41 | 28 | 28 | 28 | 28 |
| c.45 | 127 | 130 | 130 | 127 |
| c.46 | 308 | 308 | 308 | 301 |
| c.47 | 6756 | 6876 | 6912 | 6301 |
| c.50 | 6 | 6 | 6 | 6 |
| c.51 | 50 | 50 | 50 | 50 |
| c.53 | 5 | 5 | 5 | 5 |
| c.55 | 72 | 70 | 71 | 30 |
| c.56 | 205 | 205 | 205 | 203 |
| c.61 | 712 | 700 | 701 | 697 |
| c.62 | 36 | 36 | 36 | 41 |
| c.65 | 12 | 12 | 12 | 12 |
| c.66 | 6657 | 6667 | 6710 | 6617 |
| c.67 | 5387 | 5461 | 5524 | 5393 |
| c.68 | 283 | 280 | 336 | 294 |
| c.69 | 3416 | 3431 | 3431 | 3413 |
| c.71 | 42 | 42 | 42 | 38 |
| c.72 | 163 | 163 | 163 | 149 |
| c.73 | 150 | 150 | 150 | 150 |
| c.74 | 4 | 4 | 4 | 4 |
| c.78 | 11 | 11 | 11 | 11 |
| c.79 | 126 | 126 | 126 | 123 |
| c.80 | 13 | 13 | 13 | 10 |
| c.81 | 22 | 22 | 22 | 22 |
| c.90 | 30 | 30 | 30 | 30 |
| c.93 | 97 | 97 | 97 | 104 |
| c.94 | 196 | 196 | 196 | 198 |
| c.97 | 51 | 51 | 51 | 51 |
| d.101 | 60 | 60 | 60 | 60 |
| d.104 | 176 | 176 | 175 | 143 |
| d.108 | 2317 | 2317 | 2317 | 2316 |
| d.109 | 43 | 42 | 43 | 38 |
| d.113 | 836 | 836 | 840 | 838 |
| d.120 | 7 | 7 | 7 | 7 |
| d.122 | 100 | 100 | 100 | 100 |
| d.127 | 16 | 16 | 16 | 16 |
| d.129 | 65 | 62 | 79 | 65 |
| d.131 | 59 | 60 | 62 | 56 |
| d.134 | 10 | 10 | 10 | 10 |
| d.14 | 70 | 70 | 70 | 70 |
| d.142 | 115 | 115 | 115 | 121 |
| d.144 | 482 | 483 | 483 | 484 |
| d.145 | 9 | 9 | 9 | 7 |
| d.15 | 2254 | 2268 | 2306 | 1932 |
| d.153 | 37 | 37 | 37 | 37 |
| d.157 | 443 | 496 | 495 | 492 |
| d.159 | 26 | 26 | 26 | 26 |
| d.161 | 12 | 12 | 12 | 12 |
| d.166 | 18 | 18 | 18 | 18 |
| d.168 | 8 | 8 | 8 | 8 |
| d.169 | 761 | 764 | 775 | 761 |
| d.17 | 650 | 684 | 693 | 576 |
| d.185 | 70 | 70 | 70 | 82 |
| d.19 | 105 | 105 | 104 | 98 |
| d.190 | 22 | 22 | 23 | 24 |
| d.20 | 385 | 385 | 385 | 385 |
| d.21 | 11 | 11 | 11 | 6 |
| d.211 | 90 | 90 | 88 | 76 |
| d.227 | 11 | 11 | 11 | 11 |
| d.26 | 38 | 38 | 38 | 39 |
| d.287 | 4 | 4 | 4 | 4 |
| d.303 | 4 | 4 | 4 | 4 |
| d.32 | 455 | 451 | 460 | 435 |
| d.37 | 426 | 442 | 442 | 442 |
| d.38 | 1201 | 1266 | 1271 | 1385 |
| d.43 | 10 | 10 | 10 | 10 |
| d.50 | 192 | 193 | 195 | 175 |
| d.51 | 390 | 390 | 388 | 387 |
| d.54 | 234 | 234 | 234 | 227 |
| d.58 | 5988 | 6075 | 5943 | 6406 |
| d.77 | 4 | 4 | 4 | 4 |
| d.79 | 24 | 24 | 24 | 24 |
| d.82 | 3 | 3 | 3 | 3 |
| d.9 | 182 | 182 | 182 | 182 |
| d.90 | 33 | 33 | 33 | 33 |
| d.92 | 37 | 37 | 36 | 37 |
| d.93 | 576 | 576 | 576 | 574 |
