# Supplementary material for: Master Blaster: an approach to sensitive identification of remotely related proteins
Source: Sci Rep. 2021 Apr 22;11:8746. doi: 10.1038/s41598-021-87833-4 (PMC8062480; doi:10.1038/s41598-021-87833-4)
Supplement: Supplementary file 6 — Supplementary Table S5. [file 41598_2021_87833_MOESM6_ESM.docx]

**Title: Master Blaster: An approach to sensitive identification of remotely related proteins**

Authors: Chintalapati Janaki, Venkatasubramanian S. Gowri and Narayanaswamy Srinivasan

**Supplementary Table 5** - 37 SCOP Folds from which across fold connections are reported by Master Blaster (SCOP70 database) using E-value of 1e-10 and query coverage of 70%

| **S.No** | **Fold** | **SCOP ID** | **Number of across fold connections** |
| --- | --- | --- | --- |
|  | [Cytochrome c](http://scop.berkeley.edu/sunid=46625) | a.3 | 48 |
|  | EF Hand-like | a.39 | 6 |
|  | [DNA/RNA-binding 3-helical bundle](https://scop.berkeley.edu/sunid=46688) | a.4 | 4 |
|  | [RuvA C-terminal domain-like](https://scop.berkeley.edu/sunid=46928) | a.5 | 4 |
|  | Immunoglobulin-like beta-sandwich | b.1 | 2958 |
|  | [SH3-like barrel](https://scop.berkeley.edu/sunid=50036) | b.34 | 2 |
|  | [GroES-like](https://scop.berkeley.edu/sunid=50128) | b.35 | 7 |
|  | [PDZ domain-like](http://scop.berkeley.edu/sunid=50155) | b.36 | 48 |
|  | [Trypsin-like serine proteases](https://scop.berkeley.edu/sunid=50493) | b.47 | 5 |
|  | [PH domain-like barrel](https://scop.berkeley.edu/sunid=50728) | b.55 | 9 |
|  | [Cupredoxin-like](https://scop.berkeley.edu/sunid=49502) | b.6 | 6 |
|  | 6-bladed beta-propeller | b.68 | 5 |
|  | 7-bladed beta-propeller | b.69 | 4 |
|  | [Double-stranded beta-helix](https://scop.berkeley.edu/sunid=51181) | b.82 | 1 |
|  | [TIM beta/alpha-barrel](https://scop.berkeley.edu/sunid=51350) | c.1 | 7 |
|  | [HAD-like](https://scop.berkeley.edu/sunid=56783) | c.108 | 23 |
|  | NAD(P)-binding Rossmann-fold domains | c.2 | 1579 |
|  | [Flavodoxin-like](http://scop.berkeley.edu/sunid=52171) | c.23 | 2 |
|  | [FAD/NAD(P)-binding domain](http://scop.berkeley.edu/sunid=51904) | c.3 | 2850 |
|  | [DHS-like NAD/FAD-binding domain](http://scop.berkeley.edu/sunid=52466) | c.31 | 5 |
|  | [P-loop containing nucleoside triphosphate hydrolases](http://scop.berkeley.edu/sunid=52539) | c.37 | 897 |
|  | [Nucleotide-binding domain](https://scop.berkeley.edu/sunid=51970) | c.4 | 300 |
|  | [Rhodanese/Cell cycle control phosphatase](https://scop.berkeley.edu/sunid=52820) | c.46 | 1 |
|  | [Thioredoxin fold](https://scop.berkeley.edu/sunid=52832) | c.47 | 7 |
|  | [Anticodon-binding domain-like](https://scop.berkeley.edu/sunid=52953) | c.51 | 65 |
|  | [PRTase-like](https://scop.berkeley.edu/sunid=53270) | c.61 | 3 |
|  | S-adenosyl-L-methionine-dependent methyltransferases | c.66 | 1035 |
|  | [PLP-dependent transferase-like](http://scop.berkeley.edu/sunid=53382) | c.67 | 430 |
|  | [Nucleotide-diphospho-sugar transferases](https://scop.berkeley.edu/sunid=53447) | c.68 | 18 |
|  | [Alpha/beta-Hydrolases](https://scop.berkeley.edu/sunid=53473) | c.69 | 33 |
|  | [Ribokinase-like](https://scop.berkeley.edu/sunid=53612) | c.72 | 5 |
|  | [Nudix](https://scop.berkeley.edu/sunid=55810) | d.113 | 1 |
|  | Beta-Grasp (ubiquitin-like) | d.15 | 122 |
|  | [Glyoxalase/Bleomycin resistance protein/Dihydroxybiphenyl dioxygenase](https://scop.berkeley.edu/sunid=54592) | d.32 | 62 |
|  | Eukaryotic type KH-domain (KH-domain type I) | d.51 | 6 |
|  | Ferredoxin-like | d.58 | 13 |
|  | Bacillus chorismate mutase-like | d.79 | 1 |
